# circExp database: an online transcriptome platform for human circRNA expressions in cancers

**DOI:** 10.1093/database/baab045

**Published:** 2021-07-23

**Authors:** Min Zhao, Yining Liu, Hong Qu

**Affiliations:** School of Science and Engineering, University of the Sunshine Coast, Maroochydore DC, QLD 4558, Australia; The School of Public Health, Institute for Chemical Carcinogenesis, Guangzhou Medical University, Guangzhou 510182, China; Center for Bioinformatics, State Key Laboratory of Protein and Plant Gene Research, College of Life Sciences, Peking University, Beijing 100871, P.R. China

## Abstract

Circular RNA (circRNA) is a highly stable, single-stranded, closed-loop RNA that works as RNA or as a protein decoy to regulate gene expression. In humans, thousands of circRNA transcriptional products precisely express in specific developmental stages, tissues and cell types. Due to their stability and specificity, circRNAs are ideal biomarkers for cancer diagnosis and prognosis. To provide an integrated and standardized circRNA expression profile for human cancers, we performed extensive data curation across 11 technical platforms, collecting 48 expression profile data sets for 18 cancer types and amassing 860 751 expression records. We also identified 189 193 differential expression signatures that are significantly different between normal and cancer samples. All the pre-calculated expression analysis results are organized into 132 plain text files for bulk download. Our online interface, circExp, provides data browsing and search functions. For each data set, a dynamic expression heatmap provides a profile overview. Based on the processed data, we found that 52 circRNAs were consistently and differentially expressed in 20 or more processed analyses. By mapping those circRNAs to their parent protein-coding genes, we found that they may have profoundly affected the survival of 10 797 patients in the The Cancer Genome Atlas pan-cancer data set. In sum, we developed circExp and demonstrated that it is useful to identify circRNAs that have potential diagnostic and prognostic significance for a variety of cancer types. In this online and reusable database, found at http://soft.bioinfo-minzhao.org/circexp, we have provided pre-calculated expression data about circRNAs and their parental genes, as well as data browsing and searching functions.

**Database URL**: http://soft.bioinfominzhao.org/circexp/

## Introduction

Gene expression is a process of converting the information DNA encodes into functional products. Circular RNAs (circRNAs), one category of non-coding RNAs, both compete with endogenous microRNAs and modulate RNA-binding proteins and are thus important regulators at the transcriptional and post-transcriptional levels (1). Forming covalently linked continuous loops, circRNAs are abundant in the cytoplasm of eukaryotic cells and are relatively stable compared to their cognate linear forms (2). Rather than being regarded as transcriptional by-products, circRNAs are often associated with many common diseases, including human cancers (3).

The identification of molecular signatures for the diagnosis and prognosis of various cancers is difficult because of both cancer genome complexity and the enormous amount of genetic heterogeneity in human cancers. In general, and compared with circRNAs in the corresponding normal tissues, cancer-associated circRNAs are generally down-regulated and involved in tumour development, invasion, metastasis and anti-carcinogen resistance (3). These unique features make circRNAs ideal markers for diagnosing and assessing the prognoses of complex cancers. With the development of genome-wide microarray and high-throughput RNA sequencing (RNAseq) technologies, transcriptome profiling now systematically characterizes the expression changes of circRNAs. Those data sets are stored in public gene expression databases without any cross-dataset validation. Hence, our main goal here was to develop a bioinformatics tool that analyses circRNA data to identify potential signatures.

Considering the biological significance of circRNAs, integrating transcriptomes from different technical platforms would likely provide cross-validated high-quality expression events in a variety of contexts. To achieve this, we first had to standardize and annotate the circRNAs compiled in various platforms. Then, to help identify putative circRNA-related up- and down-regulated events, we developed the first free and open database, circExp (http://soft.bioinfo-minzhao.org/circexp/), that enables the characterization of circRNA expression in human cancers.

## Data collection

By advancing microarray profiling and sequencing, more and more diagnostic markers and therapeutic targets for cancer are being described, but without any cross comparison among studies (4). To explore human circRNA expression changes in cancer, we conducted extensive keyword-based searches of the public Gene Expression Omnibus (GEO) from the National Center for Biotechnology Information. By simply using ‘circRNA’ or ‘circular RNA’, we found 1103 entries, 197 of which were human data sets. We further narrowed the list to those data sets with a minimum of four samples. Finally, we manually read the data set descriptions to identify cancer-related studies, subsequently collecting 48 GEO data sets.

**Figure 1.**
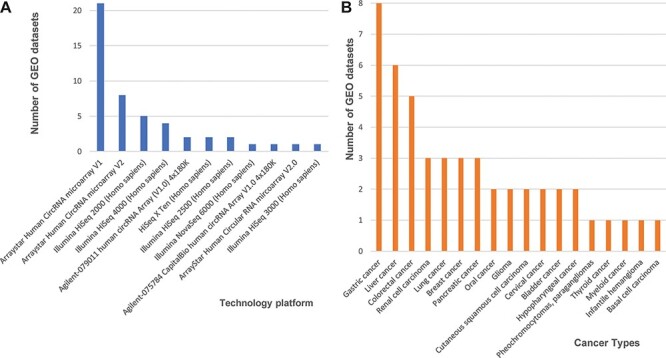
Statistics of 48 curated GEO data sets of circRNA expression in cancers. (A) The number of data sets from various technology platforms. (B) The number of data sets for curated cancer types.

Next, we found 33 microarray-based data sets, 24 from the Agilent-069978 Arraystar Human CircRNA microarray version 1 and 9 from version 2 (Figure 1A). We downloaded the array-based platforms’ information from the GEO database, and when we mapped those circRNAs back to the circRNA database IDs based on their genomic locations, we found that the total probe numbers between the Arraystar Human CircRNA microarray versions 1 and 2 differed greatly. So, we designed our data processing procedure to prefer the Arraystar Human CircRNA microarray version 2, rather than those annotations from version 1. We used circBase (5) as a reference database to annotate circRNAs consistently and then unified all probes from the Arraystar Human CircRNA microarray versions 1 and 2 into circBase IDs. Additional 14 data sets generated on Illumina high-throughput, next-generation sequencing platforms (i.e. HiSeq X Ten, HiSeq 2000, HiSeq 2500, HiSeq 3000, HiSeq 4000 and HiSeq 6000) each consisted of short reads, so we mainly used the genome to map those annotated circRNAs from circBase.

In our database, we labelled all cancer data sets with their corresponding cancer types. Three cancer types have five or more data sets that can be cross-validated, including eight data sets for gastric cancer, six for liver cancer five for colorectal cancer, and other four cancer types (renal cell carcinoma, lung cancer, breast cancer and pancreatic cancer) are associated with three independent data sets (Figure 1B). Finally, the circRNA data set summaries from different cancers and platforms enabled us to further integrate and cross-validate all 48 curated data sets.

## Web interface

**Figure 2.**
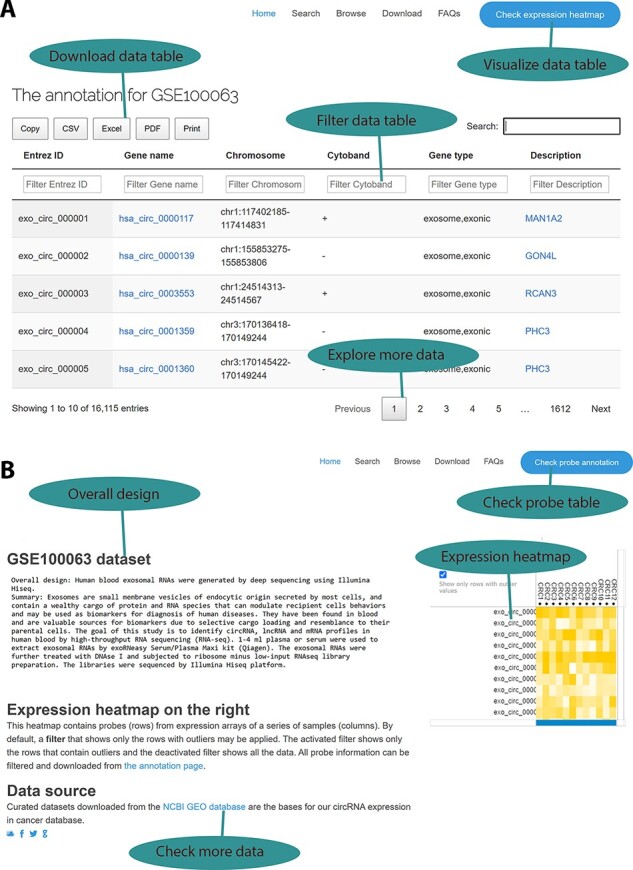
Web interface for the circExp database. (A) On the probe annotation page, users can follow four highlighted functions for data filtering, downloading and pagination. Each column in the data table can also be sorted by clicking the column number. The column filtering function works by entering a keyword for a column. The filtered results can be downloaded in Excel, CSV and PDF formats. Users can switch to the expression heatmap by clicking the button on the top right of the screen. (B) On the expression heatmap page, users find a data set’s overall design and they can link back to the original records in the public database. The data set’s heatmap explores expression patterns.

To ensure that those curated data sets and their annotations are accessible to our circRNA cancer community, we developed an online web interface for data query and browsing based on a chosen cancer type as our previous work (6). A typical data set contains two information categories: a circRNA annotation page and an expression profile page. On the circRNA annotation page (Figure 2A), users can explore all annotated information, including circRNA IDs in the circBase database, genomic location, strand, and the parental genes and their associated information. Users may load a large probe table by using the jQuery-based DataTables plug-in, which can sort, page and filter to plain HTML tables with a few Excel-like clicks. Subsequently, the resulting information can be exported in Excel, comma-separated values (CSV) and PDF formats for further data manipulation, and the handy clipboard-based copying and printing functions ensure easy data saving on local computers. On the expression profile page (Figure 2B), probe- and sample-based heatmaps change dynamically as the user scrolls down the long list of probes, thus providing a quick overview of the different expression patterns among various samples. Additionally, data set information and links to the GEO source data sets are provided. Differential expression analysis results are provided on the download page.

CircExp allows users to browse circRNA expression data based on the curated cancer types and platforms (microarray-based or RNAseq-based expression profiles) mentioned above. We constructed three text-based query interfaces that enable the user to find circRNA-associated information, experimental design and parental protein-coding genes information. For example, searching for ‘exosome’ will return all exosome-related data sets that match the experimental description in our database. All data produced by circExp (i.e. probe annotation, differential expression analysis, expression matrix and curated data set information) are shared under a Creative Commons License, so we retain the ownership of the data, although they are publicly available. While those curated and annotated data sets are useful for profile-free research, they have no warranty of merchantability for any particular purpose. To reuse our data for meta-analyses, users should start at data browsing and find multiple data sets relevant to their topics of interest. For example, we have five data sets related to colorectal cancer, likely a good starting point for a comparative analysis identifying circRNAs that are consistently up/down-regulated across multiple data sets.

## Application to identify circRNAs that expressed differently between cancer and normal samples

To develop a successful therapeutic regimen for cancer, the molecular characteristics of important driver events that cause different cancer types must be identified. Of our 48 curated data sets, 19 emphasized cancer sample profiles only, thus negating the ability to compare the differences between cancer and normal samples. The remaining 29 data sets had both cancer and normal tissue measurements. To explore the differential expression between normal and cancer tissues, we grouped those samples according to their clinical information. For example, we set up two comparisons for GSE101123. One was locally advanced breast cancer samples versus normal samples. The other comparison was for triple-negative breast cancer samples and normal samples. Similarly, there were three, two and two comparisons for GSE101124, GSE93522 and GSE93522, respectively. Therefore, we obtained 34 cancer/normal comparisons from the 29 data sets. We did so by performing differential gene expression analysis, checking each data set’s samples and separating them into cancer and normal tissue groups. For those data sets with multiple experimental conditions, we assigned the cancer samples having similar clinical features to a single cancer sample group; thus, each cancer sample will have multiple differential expression results in some circRNA cancer studies. Once the sample groups were defined, we conducted differential expression analysis using the limma package (version 3.26.8) in R (version 3.2.3). The differentially expressed circRNAs were determined by the adjust *P*-values less than 0.05. To make the expression values closer to biologically detectable changes, we used Log2 transformation to calculate fold changes, with which we then measured the up- and down-regulated genes between cancer and normal sample groups.

**Figure 3.**
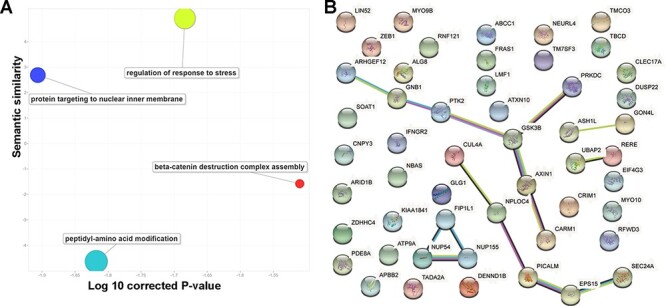
Basic functions of 52 circRNA differentially expressed parental genes found in 32 TCGA studies. (A) Key gene ontology (biological process) terms (B) The molecular network constructed from the STRING web server includes gene–gene association pairs (light green lines) gathered by text mining, gene co-expression pairs (black lines), evolutionary homolog-inferred interactions (blue lines), and large-scale experimentally validated protein–protein interactions (pink lines).

To explore the consistent circRNA changes in different cancer types, we merged all differential expression analysis results from the 29 studies. Based on the unique gene ID, we checked whether there were multiple significant differentially expressed events for the same gene in various data sets. In practice, we used ‘numbers of studies’ to prioritize important circRNA associations to cancer development and progression. Finally, we focused on 52 circRNAs with concordant differential expression across 10 cancer/normal comparisons. Since circRNA functions are normally associated with their parental protein-coding genes, we mapped those 52 circRNAs to their coding genes and conducted a literature similarity search and a functional analysis. While inspecting the associated biological processes (Figure 3A), we found 14 genes involved in regulating the stress response (False discovery rate (FDR)-corrected *P*-value = 2.07E-2) and 13 involved in peptidyl-amino acid modification (False discovery rate (FDR)-corrected *P*-value = 1.52E-2). The literature similarity search revealed 52 maternal genes that were linked to cancer in various ways. For example, 19 genes that act in Nerve growth factor (NGF)-Tropomyosin receptor Kinase A (TrkA) signalling were found to interact with neurotrophic receptor tyrosine kinase 1 (NTRK1) during neuroblastoma cell differentiation (FDR-corrected *P*-value = 1.59E-8) and that the same kinase is connected to transcriptional misregulation in cancer. Additionally, cells with one or more of the 15 overrepresented genes in the down-regulated gene list for breast cancer undergo apoptosis in response to doxorubicin (FDR-corrected *P*-value = 1.59E-8).

Examination of subcellular localization revealed nine genes located on the ER membrane (FDR-corrected *P*-value = 3.08E-2) through the nuclear outer membrane–endoplasmic reticulum (ER) membrane network and another five from the lamellipodium (FDR-corrected *P*-value = 2.94E-2). Both ER and lamellipodia have important roles during cancer development. As dynamic extensions on the cell surface, lamellipodia are involved in regulating cancer cells’ migratory and invasive abilities. Combining genomic location, gene fusion, co-occurrence, co-expression and text mining evidence from the STRING database (version 11.0), we found that 19 of the 52 parental genes could be connected to each other (Figure 3B). For example, GSK3B and AXIN1 form a molecular axis in the Wnt signalling pathway and, based on thousands of The Cancer Genome Atlas (TCGA) expression profiles, they are highly correlated (Supplementary Figure S1). More interestingly, those genes are found in the Wnt-Frizzled-LRP5/6 complex in the Wnt signalosome. Since they are highly correlated with patient survival, the remaining 33 isolated circRNA parental genes may be used to explore potential novel mechanisms in multiple cancer types.

## Linking circRNA parental genes to phenotypes by overlapping to genetic variant databases

To explore the 52 differentially expressed genes, we used cBioPortal to check those genes’ mutational patterns in the TCGA pan-cancer data set (7) and then used OncoPrint to create visualizations of sample-based mutational patterns from 10 967 tumour samples taken from 10 953 patients investigated in 32 TCGA studies. Providing a comprehensive overview of mutational patterns meant that cBioPortal included all single nucleotide variations, gene fusions and copy number variations—all important factors in cancer development and progression (8–10).

**Figure 4.**
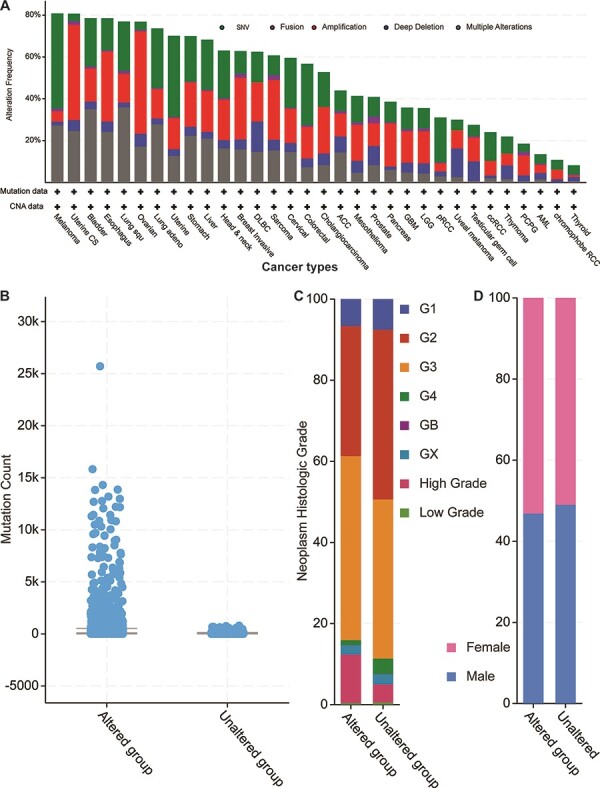
Cancer genomics overview of the 52 circRNA differentially expressed parental genes in 32 TCGA studies. (A) Mutational frequencies in various cancer types. (B) The mutation counts for those samples with (altered) or without (unaltered) genetic variants among the 52 genes. (C) The histological grade distributions for those samples with or without genetic variants among the 52 genes. (D) Gender ratios for those samples with or without genetic variants among the 52 genes.

Next, we explored the 52 genes’ genetic features by first mapping those genes to the TCGA pan-cancer data set mentioned above (Figure 4). By checking all possible genetic changes, we found that those genes were altered in 5928 (54%) of the 10 953 patients. The alteration frequencies of two cancer types (melanoma and uterine cancer) exceeded 80%, meaning that over 80% of the patients had genetic changes in one or more of the 52 genes (Figure 4A). In other 13 studies of TCGA bladder, oesophagus, lung squamous cell carcinoma, ovarian, lung adenocarcinoma, uterine, stomach, liver, head and neck, breast invasive, diffuse large B-cell lymphoma, sarcoma and cervical cancers, the mutational frequencies were all greater than 60%. Overall, our 52 parental genes were highly mutated in thousands of samples, thus indicating potentially common mechanisms among different cancer types.

When examining total genetic mutations, we found that the mutation counts between the altered and unaltered sample groups described above differed greatly (Figure 4B). Also, the altered group’s samples were relatively high-grade compared to those of the unaltered group’s (Figure 4C), but the male/female distributions were similar within each sample group (Figure 4D). The most mutated gene was Protein tyrosine kinase 2 (*PTK2*) (Supplementary Figure S2), which was mutated in 813 cancer samples in multiple studies (7.4% of the 10 967 samples). Another five genes (Protein kinase, DNA-Activated, catalytic subunit (*PRKDC*), ASH1 like histone lysine methyltransferase (*ASH1L*), gon-4 like (*GON4L*), myosin X (*MYO10*) and Fraser extracellular matrix complex subunit 1 (*FRAS1*)) are mutated in over 5% of those samples. Notably, while the circRNA of *PKT2* has been reported to modify radiosensitivity in gastric cancer via the miR-369-3p/Zinc finger E-box Binding homeobox 1 (ZEB1) regulatory motif (11), circ-PRKDC regulates the miR-375/Forkhead box M1 (FOXM1) axis and the *Wingless and Int1* (Wnt)/β-catenin pathway in colorectal cancer cells, thus contributing to chemotherapy drug resistance (12). Given the parental coding genes’ high mutational frequencies in the pan-cancer studies, our analyses suggest that their circRNAs may have similar roles in multiple cancer types.

**Figure 5.**
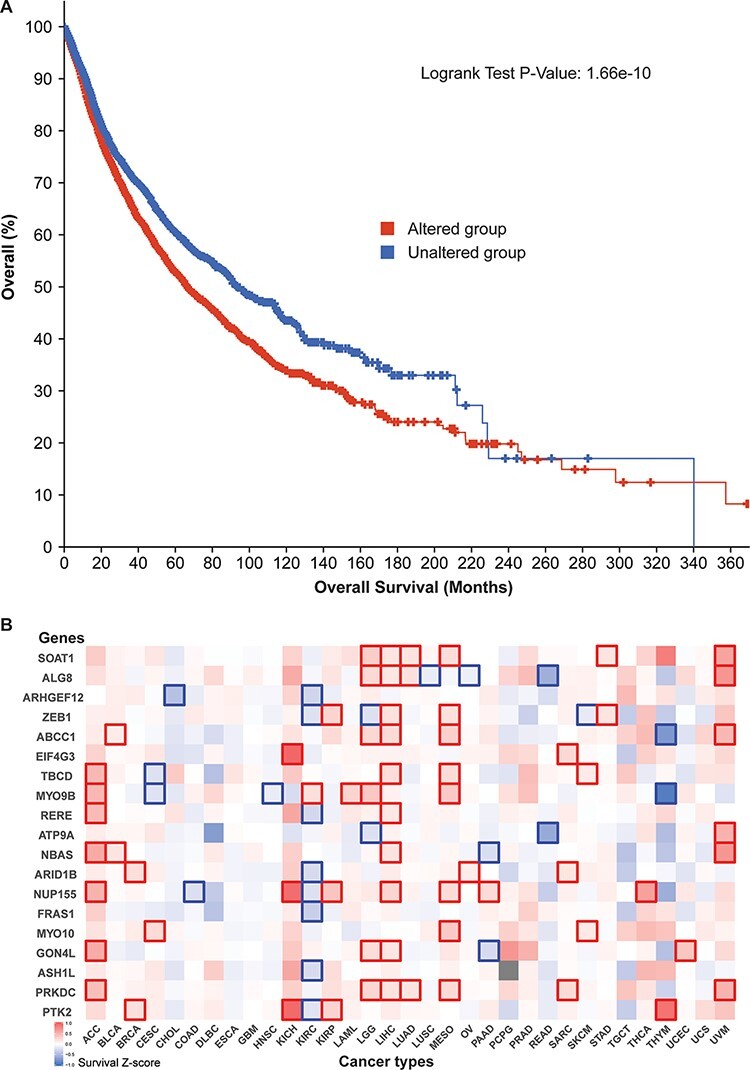
Cancer patient survival with regard to the 52 circRNA parental genes. (A) Overall survival analysis of 10 797 patients in our TCGA pan-cancer data set. See Figure 4 for definitions of altered and unaltered. (B) Cancer-type-specific survival analysis. *Z*-score polarities reflect the genes’ beneficial or detrimental effects on survival. Bolded squares highlight statistically significant survival differences.

Additionally, survival analysis of 10 797 patients revealed that our 52 parental genes aid cancer prognoses. Of the 5839 cases with genetic mutations among those 52 genes, median survival (66.64 months) was significantly lower than that of the unaltered group (93.89 months) (Figure 5A). By zooming in different cancer types, we prepared a survival significance map for 19 genes that had mutational frequencies of 3% or higher in the 10 797 patients (Figure 5B). For example, *PTK2* figured significantly in breast cancer (BRCA), three kidney cancer data sets [kidney chromophobe (KICH), kidney renal clear cell carcinoma (KIRC) and kidney renal papillary cell carcinoma (KIRP)], and thymoma (THYM), while *PRKDC* has distinct patterns in seven cancer studies. Since circRNAs have many regions that overlap their corresponding protein-coding transcripts, those mutation-based analyses indicate that those related circRNAs may have profound effects on patient survival.

## circRNA variant types in the human genome

**Figure 6.**
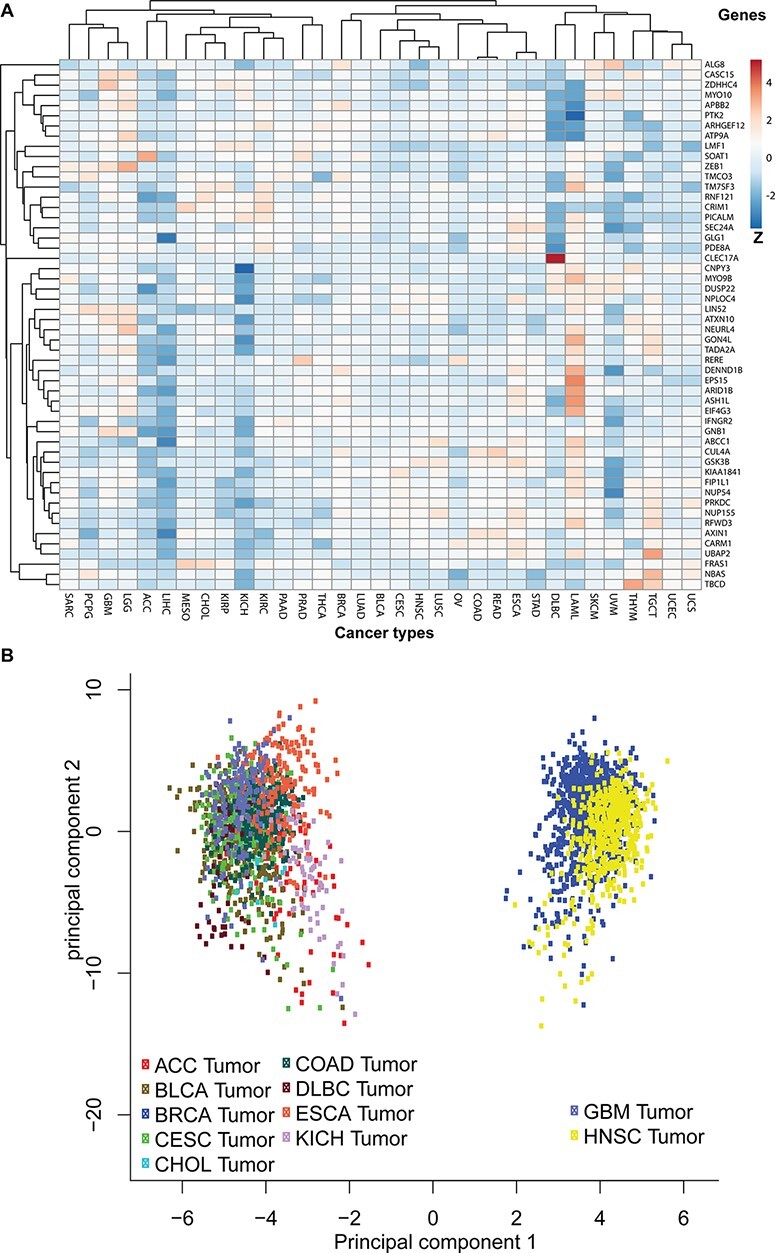
Pan-cancer expression pattern analyses. (A) The pan-cancer expression patterns for the 52 circRNA parental genes. Genes with similar expression patterns are clustered together. The *Z*-scores indicate higher or lower than average expressions in each category. (B) A principal component analysis based on pan-cancer expression profiles. The TCGA data sets are plotted in different colours: adrenocortical carcinoma (ACC), bladder urothelial carcinoma (BLCA), breast invasive carcinoma (BRCA), cervical squamous cell carcinoma and endocervical adenocarcinoma (CESC), cholangiocarcinoma (CHOL), colon adenocarcinoma (COAD), oesophageal carcinoma (ESCA), glioblastoma multiforme (GBM), head and neck squamous cell carcinoma (HNSC) and KICH.

To discover the likelihood that the 52 parental genes are functionally linked to each other, we first mapped them to the TCGA pan-cancer expression profile and then plotted their average expressions in each cancer type (Figure 6A). We also clustered genes with similar expression patterns across 33 studies. Additionally, principal component analysis revealed that gene expressions of those 52 coding genes clustered certain cancer types into two separate groups (Figure 6B). One group includes glioblastoma and head and neck cancer and the other includes nine other cancer types, such as bladder, breast, colorectal and kidney cancers.

The preceding group of 52 circRNAs were based on an integrative analysis of all 29 differential expression data sets. If we zoom in on a specific cancer type in our circExp database, we may identify novel circRNA-associated mechanisms. In summary, our huge number of expression profiles and differential expression data aid the exploration of novel mechanisms associated with circRNA expressions that may influence cancer patients’ lives, even after treatments have ceased.

## Conclusion

To provide a source of integrated and standardized circRNA expression profiles in human cancers, we performed extensive data curation and provided consistent annotations across multiple technical platforms and developed circExp. The formatted data in our circExp database contains (i) 48 curated data sets with phenotype information from genome-wide expression profiles in various cancer types, (ii) 189 193 pre-calculated differentially expressed events and (iii) 860 751 expression records across multiple technical platforms The circExp web interface allows users to perform text queries and browse circRNAs based on their parental genes and cancer types. For advanced bioinformatics analysis, we provided 132 bulk downloadable files of all circRNAs including 1 data set summary table, 48 annotated probe datasheets, 49 expression matrix files (GSE93522 has two matrix files) and 34 differential expression comparison results. Additionally, our integrative analysis of the 52 shared, differentially expressed genes among 29 data sets makes our database a useful tool with which to explore circRNA regulatory mechanisms.

## Supplementary Material

baab045_Supp

## Data Availability

All the data are free to use for academic purpose at http://soft.bioinfo-minzhao.org/circexp.
