# Supplementary figures and images for: circExp database: an online transcriptome platform for human circRNA expressions in cancers
Source: Database (Oxford). 2021 Jul 23;2021:baab045. doi: 10.1093/database/baab045 (PMC8299715; doi:10.1093/database/baab045)

p-value =  $2.7\text{e-}300$

R = 0.36

log<sub>2</sub>(AXIN1 TPM)

6  
5  
4  
3  
2  
1  
0

log<sub>2</sub>(GSK3B TPM)

0 1 2 3 4 5 6 7

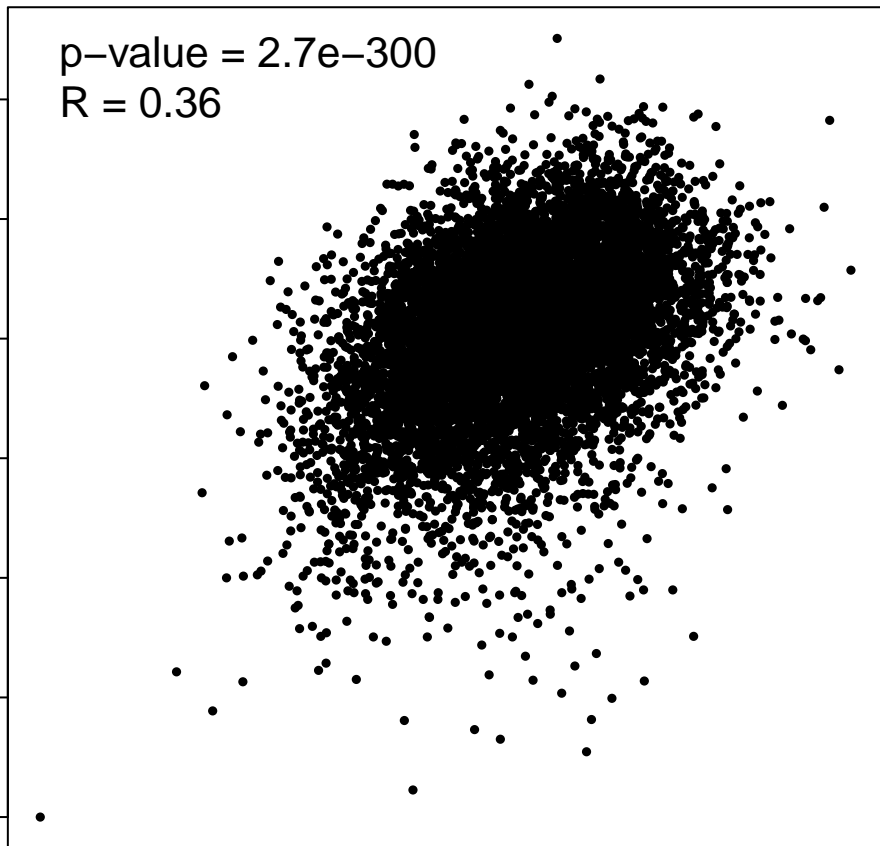

Supplement: baab045_Supp [file baab045_supp.zip › FigureS1_GSK3B_AXIN1_correlation_tumor_sample_only.pdf]

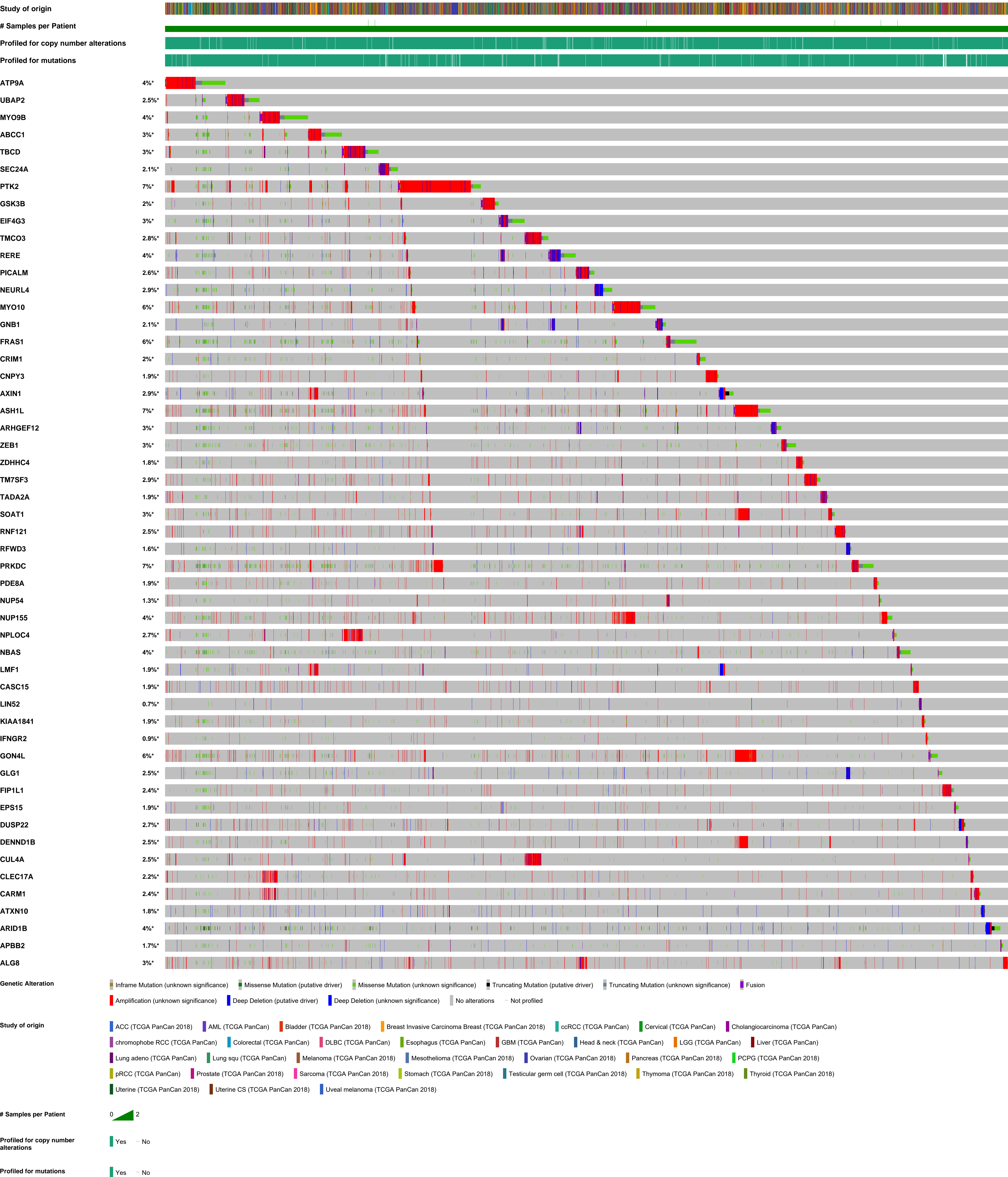

Supplement: baab045_Supp [file baab045_supp.zip › FigureS2_oncoprint.pdf]
